# Genome-Wide Histone H3K27 Acetylation Profiling Identified Genes Correlated With Prognosis in Papillary Thyroid Carcinoma

**DOI:** 10.3389/fcell.2021.682561

**Published:** 2021-06-11

**Authors:** Luyao Zhang, Dan Xiong, Qian Liu, Yiling Luo, Yuhan Tian, Xi Xiao, Ye Sang, Yihao Liu, Shubin Hong, Shuang Yu, Jie Li, Weiming Lv, Yanbing Li, Zhonghui Tang, Rengyun Liu, Qian Zhong, Haipeng Xiao

**Affiliations:** ^1^Department of Endocrinology, The First Affiliated Hospital, Sun Yat-sen University, Guangzhou, China; ^2^Zhongshan School of Medicine, Sun Yat-sen University, Guangzhou, China; ^3^Sun Yat-sen University Cancer Center, State Key Laboratory of Oncology in South China, Collaborative Innovation Center for Cancer Medicine, Guangzhou, China; ^4^Institute of Precision Medicine, The First Affiliated Hospital, Sun Yat-sen University, Guangzhou, China; ^5^Clinical Trials Unit, The First Affiliated Hospital, Sun Yat-sen University, Guangzhou, China; ^6^Department of Breast and Thyroid Surgery, The First Affiliated Hospital, Sun Yat-sen University, Guangzhou, China

**Keywords:** papillary thyroid carcinoma, benign thyroid nodule, enhancer, H3K27ac, transcriptome, epigenetics

## Abstract

Thyroid carcinoma (TC) is the most common endocrine malignancy, and papillary TC (PTC) is the most frequent subtype of TC, accounting for 85–90% of all the cases. Aberrant histone acetylation contributes to carcinogenesis by inducing the dysregulation of certain cancer-related genes. However, the histone acetylation landscape in PTC remains elusive. Here, we interrogated the epigenomes of PTC and benign thyroid nodule (BTN) tissues by applying H3K27ac chromatin immunoprecipitation followed by deep sequencing (ChIP-seq) along with RNA-sequencing. By comparing the epigenomic features between PTC and BTN, we detected changes in H3K27ac levels at active regulatory regions, identified PTC-specific super-enhancer-associated genes involving immune-response and cancer-related pathways, and uncovered several genes that associated with disease-free survival of PTC. In summary, our data provided a genome-wide landscape of histone modification in PTC and demonstrated the role of enhancers in transcriptional regulations associated with prognosis of PTC.

## Introduction

Thyroid carcinoma (TC) is the most common endocrine malignancy with an increasing incidence during the past decades (Kilfoy et al., 2009). Papillary TC (PTC) is the most frequent subtype, which accounts for 85–90% of all the TC cases and occurs three times more frequently in women than in men (Jemal et al., 2010). It is now well accepted that both genetic alterations and epigenetic changes contribute to PTC development and progression. Some somatic driver mutations, such as the *BRAF* V600E and *TERT* promoter mutations (Liu et al., 2017), and epigenetic alterations, such as specific non-coding RNAs and DNA methylation modifications (Yu et al., 2012; Yim et al., 2019), have been well established as diagnostic and prognostic markers of PTC.

Histones are subject to a variety of post-transcriptional modifications including methylation, acetylation, phosphorylation, and ubiquitination (Bártová et al., 2008; Matsuda et al., 2015), which influence interactions between DNA and histones, resulting in global regulation of gene expression. Typically, histone acetylation is related to active transcription, while deacetylation induced transcriptional silencing. Histone modifications control gene expression, and numerous studies have found that dysregulation of this modification functionally impacts transcriptome in carcinogenesis. Several histone deacetylated inhibitors such as vorinostat (SAHA), a suberoylanilide hydroxamic acid that inhibits deacetylase enzymatic activity, had been used in clinical trials treating TC patients. Moreover, it was reported that SAHA induced apoptosis in PTC cell lines (Brest et al., 2011). Recently, one study reported global levels of histone modifications in four thyroid tissues (Siu et al., 2017). Studies reported histone acetylation increment at the promoters of *NIS* and *ECAD* in TC cells (Federico et al., 2009; Zhang et al., 2014).

It is now recognized that enhancers and super-enhancers (SEs) are crucial components of genetic transcription regulators in cancers. Enhancer, a DNA region that transcription factors can bind, could positively regulate gene expression in *cis-* or *trans-* manner. It has been proposed that tumorigenesis is usually accompanied by dysregulation of enhancer activities (Bulger and Groudine, 2011). SEs, defined as clusters of enhancers densely occupied with mediators and chromatin regulators, can facilitate expressions of important genes in determining cell identity and fate (Whyte et al., 2013). Characterized by high levels of H3K27ac, enhancers can be readily identified by chromatin immunoprecipitation followed by deep-sequencing (CHIP-seq). Moreover, SEs are more prone to perturbations than typical enhancers (Zhou et al., 2015). One of the mechanisms that histone acetylation mediates RNA transcriptions is to interact with the bromodomain and extra-terminal domain (BET) protein BRD4 (Dey et al., 2003), which could serve as potential therapeutic targets of cancers. BET inhibitor JQ1 inhibits interactions between BET protein and acetylated histones, resulting in downregulation of a number of genes and related signaling pathways (Filippakopoulos et al., 2010). It has been reported that JQ1 inhibited the tumor growth in differentiated and undifferentiated TC (Gao et al., 2016; Zhu et al., 2017).

Accumulating evidence underscored the role of enhancer-driven transcriptional programs in tumor pathogenesis. However, the H3K27ac landscape and enhancer pattern of PTC remained unclear. Here, we generated reference epigenome data for PTC and benign thyroid nodule (BTN) tissues using H3K27ac ChIP-seq along with global transcriptome, comparing epigenomic features and SEs between these two groups. This study provided an epigenetic insight for understanding the development of PTC, revealing regulations of epigenomic modifications on the transcription level, and identified several enhancer-regulated genes that hold the potential to serve as diagnostic and prognostic markers for PTC.

## Materials and Methods

### Thyroid Tissues

This study included eight PTC and four BTN female cases who were treated at The First Affiliated Hospital of Sun Yat-sen University. The diagnosis of PTC was performed according to the WHO criteria. The thyroid specimens and clinicopathologic data were collected after our institutional review board approval.

### H3K27ac ChIP-seq and Library Preparation

Primary PTC and BTN were pulverized in liquid nitrogen with mortar. Tissue powder was then cross-linked with 1% formaldehyde and lysed with lysis buffer. Genomic DNA was then sonicated into 200 to 500 bp fragments using ultrasonicator in lysis buffer. The supernatant was diluted, and the lysates were incubated with anti-H3K27ac antibody (ab4729; Abcam, Cambridge, United Kingdom) overnight at 4°C. Protein–DNA complexes were captured with protein A agarose beads. After extensive washing, protein–DNA complexes were eluted and reverse cross-linked. DNA was purified with QIAGEN (Hilden, Germany) PCR purification kit (Cat No. 28106). Ten nanograms of purified DNA was used for downstream library-prep with NEBNext Ultra II DNA Library Prep Kit for Illumina (NEB #E7103; New England Biolabs, Ipswich, MA, United States), following the manufacturer’s instructions. The quality of sequencing libraries was analyzed with bioanalyzer (Agilent Technologies, Santa Clara, CA, United States) and then sequenced by Illumina NovaSeq 6000 platform. Input DNA from each sample were sequenced using different bar codes.

### Processing of Raw ChIP-seq Data

The clean ChIP-seq reads were aligned to the human genome (hg38) using BWA with default parameters. Then, the unmapped reads and non-uniquely mapped reads (mapping quality < 20) were removed by SAMtools, while the PCR duplicate reads were filtered by Picard. The H3K27ac modification regions were defined using MACS. To find out the differential H3K27ac modification region, the modification regions were merged by BEDtools, and the reads of the merged regions were estimated by HTSeq. Finally, DESeq2 was performed to normalize the counts and to detect differential H3K27ac regions (| fold change| ≥ 2 and *p* value < 0.05).

### RNA-seq and Library Preparation

Primary tissues were pulverized in liquid nitrogen, and RNA was extracted with TRIzol (Invitrogen, Carlsbad, CA, United States) following manufacturer’s protocol. RNA-seq was performed by Berry Genomics Co., China. Briefly, RNA degradation and contamination were monitored on 1% agarose gels, and purity was checked with NanoPhotometer spectrophotometer (IMPLEN, Westlake Village, CA, United States). RNA integrity was assessed with RNA Nano 6000 Assay Kit of the Bioanalyzer 2100 system (Agilent Technologies, Santa Clara, CA, United States). A total amount of 1 μg of RNA per sample was used as input material for the RNA preparations. Sequencing libraries were generated with NEBNext Ultra RNA Library Prep Kit for Illumina (NEB, United States) following manufacturer’s recommendations, and index codes were added to attribute sequences to corresponding sample. Library quality was assessed on the Agilent Bioanalyzer 2100 system. After cluster generation, the library preparations were sequenced on an Illumina NovaSeq 6000 platform.

### Processing of Raw RNA-seq Data

Differential expression analysis was performed with DESeq; the clean RNA-seq reads were aligned to the human genome (hg38) using HISAT with default parameters. Then, the reads overlapped with genes were estimated by HTseq. The normalization, counts, and detection of differentially expressed genes were performed by DEseq2 (| fold change| ≥ 2 and *p* value < 0.05).

### Pathway Enrichment Analysis

Genomic Regions Enrichment of Annotations Tool (GREAT)^1^ was used to analyze the functional significance of differentiated H3K27ac-modified regions (McLean et al., 2010). Pathway analysis was performed with gene names using DAVID. Selected Kyoto Encyclopedia of Genes and Genomes (KEGG) pathways that have *p* values of less than 0.05 were reported. Pathway enrichment analysis was performed using gene set enrichment analysis (GSEA)^2^ (Liao et al., 2019).

### Identification of Enhancer

Enhancers and promoters were identified by MACS (Liu, 2014). To identify SEs, all enhancers were ranked according to their total ChIP-seq signal using ROSE (Zhou et al., 2015). Enhancers were sorted and plotted based on H3K27ac signals in ascending order. The cutoff value was set to distinguish SEs from typical enhancers, in which enhancers were assigned as SEs if their H3K27ac signals exceeded this threshold level.

### Cell Culture

BCPAP and KTC-1 cells were cultured in RPMI-1640 media supplemented with 10% fetal bovine serum (FBS; #10270-106; Gibco, Thermo Fisher Scientific, Waltham, MA, United States). TPC-1 cells were cultured with Dulbecco modified Eagle medium supplemented with 10% FBS (Gibco). All the cells were maintained at 37°C in a 5% CO_2_ humidified chamber.

### SAHA/JQ1 Treatment

BCPAP, KTC-1, and TPC-1 cells were seeded on day 0 and treated with JQ1 (S7110; Selleck, Houston, TX, United States) or Vorinostat (SAHA) (S1047; Selleck) or vehicle for 48 h with indicated concentrations on day 1. Fresh culture medium with drugs or vehicle was replenished every 24 h.

### qRT-PCR

Total mRNAs were extracted using RNAsimple Total RNA Kit (#DP419; TIANGEN Biotech, Beijing, China). Five hundred nanograms of mRNA was used as template for reverse transcription with PrimeScript RT Master Mix (RR036A; Takara, Dalian, China). cDNAs were then amplified on ABI QuantStudio 5 Real-Time PCR System (Thermo Fisher), and SYBR Green Master Mix (A25742; Thermo Fisher Scientific, Waltham, MA, United States) was used to detect cDNA amplification. GAPDH was used to normalize gene expression. RNA relative expression was calculated using the 2^–ΔΔCT^ method.

### Statistical Analysis

Statistical analyses were performed using R and GraphPad Prism software. Student’s *t*-test was performed to compare two groups of the continuous variables. All the *p* values were two-sided, and *p* < 0.05 was regarded as statistically significant.

## Results

### Epigenetic Landscapes of Papillary Thyroid Carcinoma and Benign Thyroid Nodule

**FIGURE 1:**
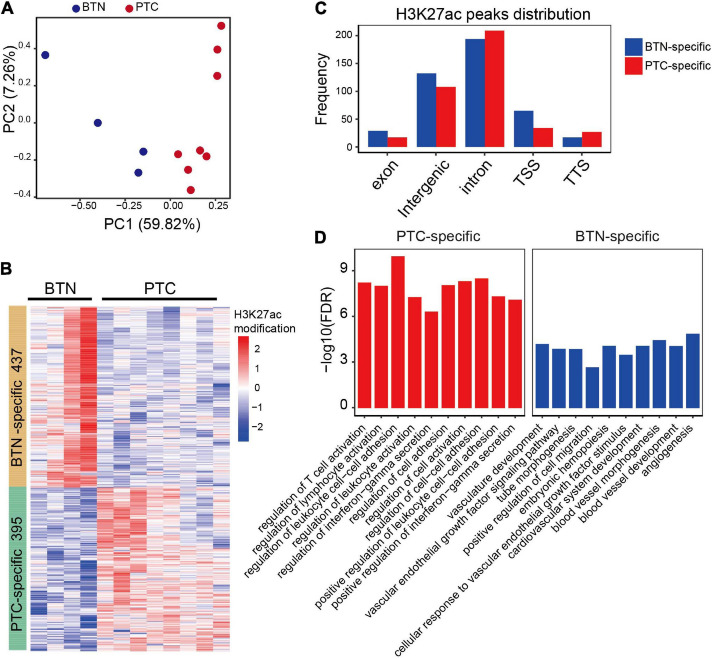
Characterization of H3K27ac landscapes in PTC and BTN. **(A)** Principal component analysis of normalized PTC and BTN H3K27ac peaks. **(B)** Heatmap of differentiated H3K27ac-modified peaks between PTC and BTN. **(C)** Distribution of differentiated histone H3K27ac marks in genome. TSS, transcription start sites; TTS, transcription termination sites. **(D)** Bar plots showing top gene ontology (GO) terms of PTC and BTN-specific H3K27ac peaks. PTC, papillary thyroid carcinoma; BTN, benign thyroid nodule.

To identify histotype-specific landscapes of active chromatin in thyroid tissues, ChIP-seq using H3K27ac (acetylated lysine 27 of histone H3) antibody was performed on eight PTC and four BTN samples from 12 Chinese females (Supplementary Data). Model-based analysis for ChIP-seq (MACS) was used to analyze and identify significant peaks from the samples. Principal component analysis (PCA) indicated that PTC and BTN samples grouped into two clusters according to their genome-wide H3K27ac profiles, suggesting that the differences were etiology specific (Figure 1A). When comparing H3K27ac peaks between PTC and BTN, 395 peaks were unique to PTC samples, while 437 peaks were unique to BTN. With all the differentiated peaks, PTC and BTN samples segregated well on hierarchical clustering (Figure 1B). Differentiated H3K27ac peaks distributed across different regions genome-wide, which showed a greater distribution in intron and intergenic regions with 8.6% PTC-specific and 14.8% BTN-unique peaks located at promoter–transcription start site (TSS) regions (Figure 1C). After the differentiated peaks were annotated with GREAT, we found that gene ontology (GO) terms enriched with PTC-specific peaks included T-cell activation, regulation of lymphocyte activation, leukocyte cell–cell adhesion, and interferon-gamma secretion. Vasculature development, vascular endothelial growth factor signaling pathway, tube morphogenesis, etc., were enriched with BTN-unique peaks (Figure 1D).

### Super-Enhancer-Regulated Genes Differed in Papillary Thyroid Carcinoma and Benign Thyroid Nodule

**FIGURE 2:**
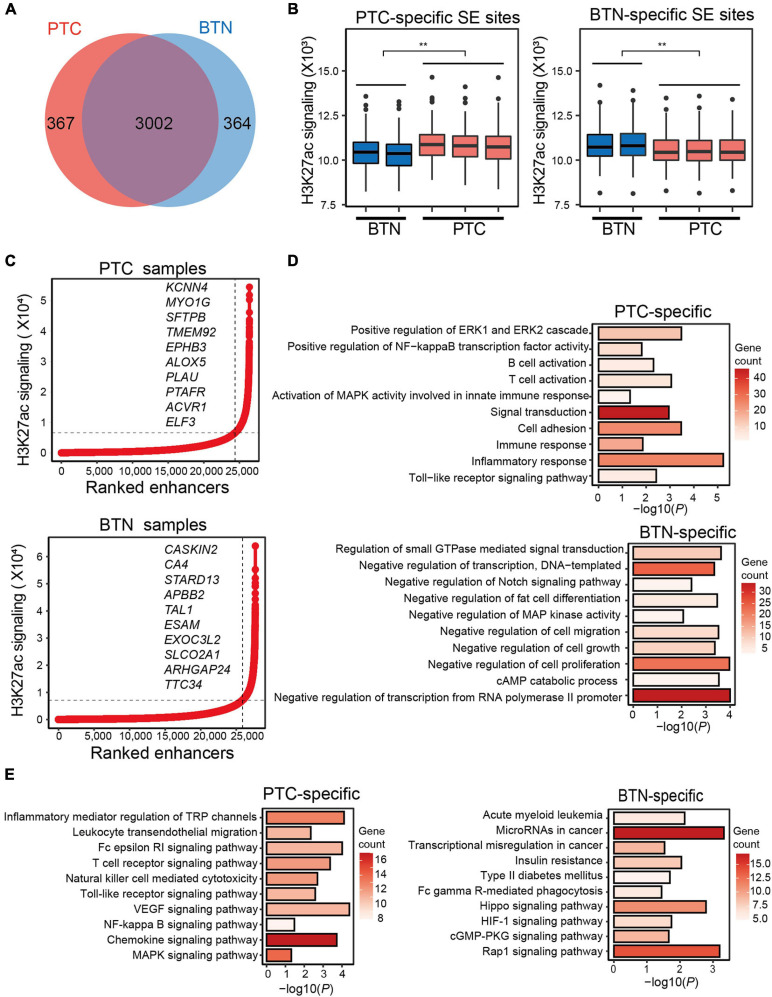
Characterization of super-enhancers in PTC and BTN. **(A)** Venn diagram showing SE numbers of PTC-specific, BTN-specific, and overlapped SEs. **(B)** Histotype-specific genomic SEs showed significantly different H3K27ac signaling levels at PTC-specific or BTN-specific SE genomic sites. **p < 0.01. **(C)** Enhancers of representative PTC and BTN sample were ranked by their H3K27ac signaling. Important genes were indicated by their ranks. **(D)** Bar plots showing GO terms of PTC- and BTN-specific SE-associated genes. **(E)** Bar plots showing KEGG pathway analysis in PTC and BTN. PTC, papillary thyroid carcinoma; BTN, benign thyroid nodule; SE, super-enhancer; GO, gene ontology; KEGG, Kyoto Encyclopedia of Genes and Genomes.

Super-enhancers are clusters of enhancers and are critically important in development, differentiation, and oncogenesis (Whyte et al., 2013). To identify SEs, H3K27ac signals from sliding windows containing enhancers were ranked using ROSE (Zhou et al., 2015). We first identified the SEs in the tumor and BTN tissues, and enhancers in each sample were annotated (Supplementary Data). A total of 3,369 SEs were identified in PTC and 3,366 SEs in BTN samples, among which 367 and 364 SEs were specific to PTC and BTN, respectively (Figure 2A). SE genomic sites were categorized into PTC-specific and BTN-specific SE sites. H3K27ac signaling of BTN SEs at PTC-specific genomic sites was significantly lower than that of PTC, contrary to the situation at BTN-specific SE genomic sites (Figure 2B).

Enhancers were ranked based on their H3K27ac signals (Figure 2C). Some SE-adjacent genes in PTC were known to be involved in TC or play a part in oncogenesis, such as *EPHB3*, *ALOX5*, and *ELF3.* In contrast, SEs in BTN included some tumor suppressor genes, which were previously reported in various cancers, such as *ARHGAP24* and *CA4.* SEs were assigned to their putative target genes and were then subjected to pathway enrichment analysis to determine their biological functional classifications (Supplementary Data). As is shown in GO analyses, these SE-adjacent genes of PTC uncovered some immune-related pathways such as immune cell activation and immune response, and other related pathways included positive regulation of ERK1 and ERK2 cascades, and regulation of NF-kappa B transcription factor activity. BTN-specific GO terms revealed pathways of negative regulation of transcription, cell migration, cell growth, etc. (Figure 2D). KEGG pathway analysis indicated some immune-related and cancer-related pathways in SE-adjacent genes of PTC (Figure 2E).

### Transcriptome Features of Papillary Thyroid Carcinoma and Benign Thyroid Nodule

**FIGURE 3:**
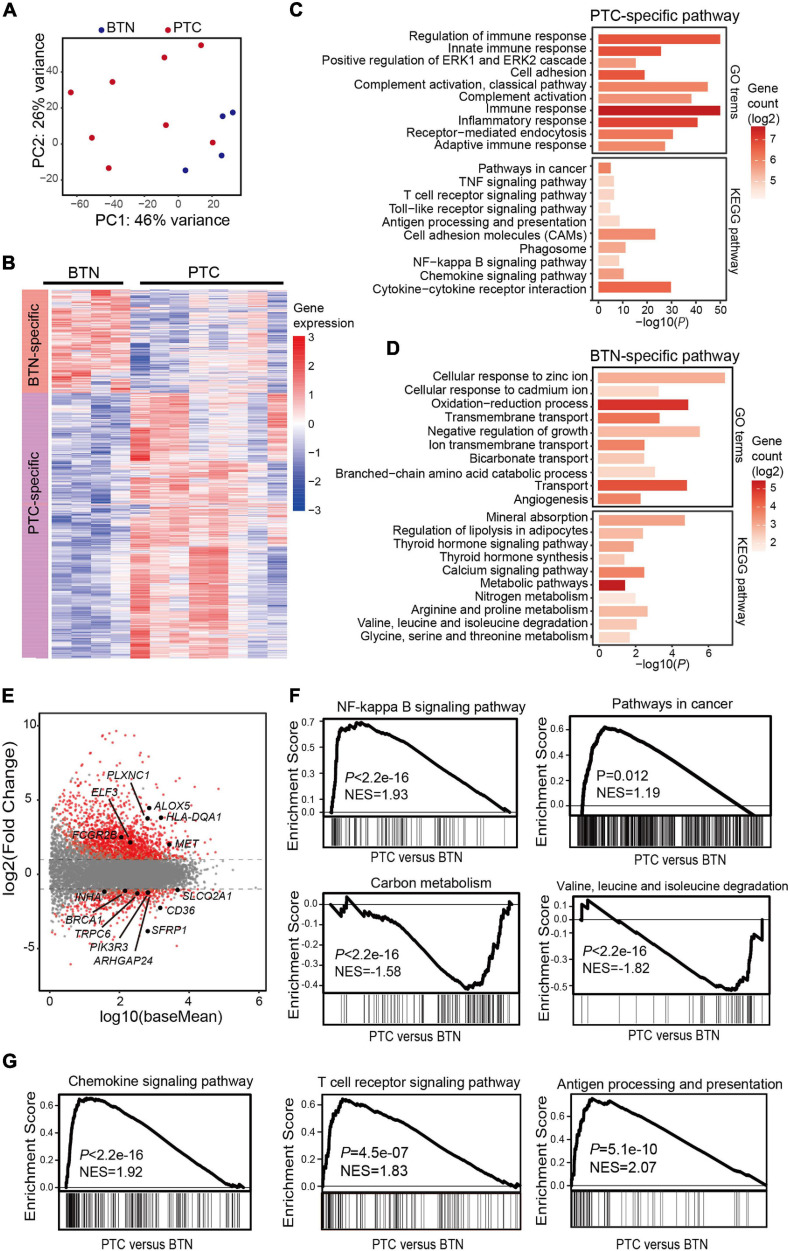
Transcriptome characterization of PTC and BTN tissues. **(A)** Principal component analysis of transcriptomes in PTC and BTN. **(B)** Heatmap of gene expression values in PTC and BTN. **(C,D)** Enrichment analysis for GO terms and KEGG pathway. Top pathways for PTC-upregulated and BTN-upregulated genes are shown. **(E)** MA plot for differentiated analysis of gene expression between PTC and nodule tissues. The red plots at the upper side of the line represent PTC-specific genes, while plots at the opposite side represent nodule-specific genes. **(F)** GSEA plots indicated cancer-related and metabolism-related genes in PTC and BTN samples. **(G)** GSEA plots showed PTC-upregulated genes enriched in immune-related pathways. PTC, papillary thyroid carcinoma; BTN, benign thyroid nodule; GO, gene ontology; KEGG, Kyoto Encyclopedia of Genes and Genomes; GSEA, gene set enrichment analysis.

Global transcriptomic features were analyzed in thyroid samples, and histotype-specific patterns of gene expression for the two groups were identified. There were 2,019 differentially expressed genes specific to PTC, while 800 genes showed a higher expression in BTN (Figures 3A,B and Supplementary Data). KEGG enrichment analysis of differentiated genes revealed that genes with higher expressions in PTC were mostly enriched in immune functions such as T-cell receptor signaling pathway, chemokine signaling pathway, antigen processing, and presentation, while BTN-specific genes were over-represented in Rap1 signaling pathway, calcium signaling pathway, mineral absorption, etc. GO annotations also revealed that genes with a higher expression in PTC were mainly involved in pathways such as immune response and inflammatory response. In contrast, BTN samples had a higher expression in metabolism pathways such as oxidation-reduction process, cellular response to hypoxia, and cellular response to zinc ion (Figures 3C,D and Supplementary Data).

The differentiated genes between the two groups were depicted (Figure 3E). Some known oncogenes and tumor suppressor genes were found in PTC-specific or BTN-specific genes, respectively (Figure 3E). GSEA was conducted and showed significant enrichment of PTC-specific genes in cancer-related pathways, such as NF-kappa B, while BTN-specific genes were enriched in metabolism-related pathways (Figure 3F). Besides, GSEA indicated enrichment of immune-related pathways in PTC-specific genes (Figure 3G).

**FIGURE 4:**
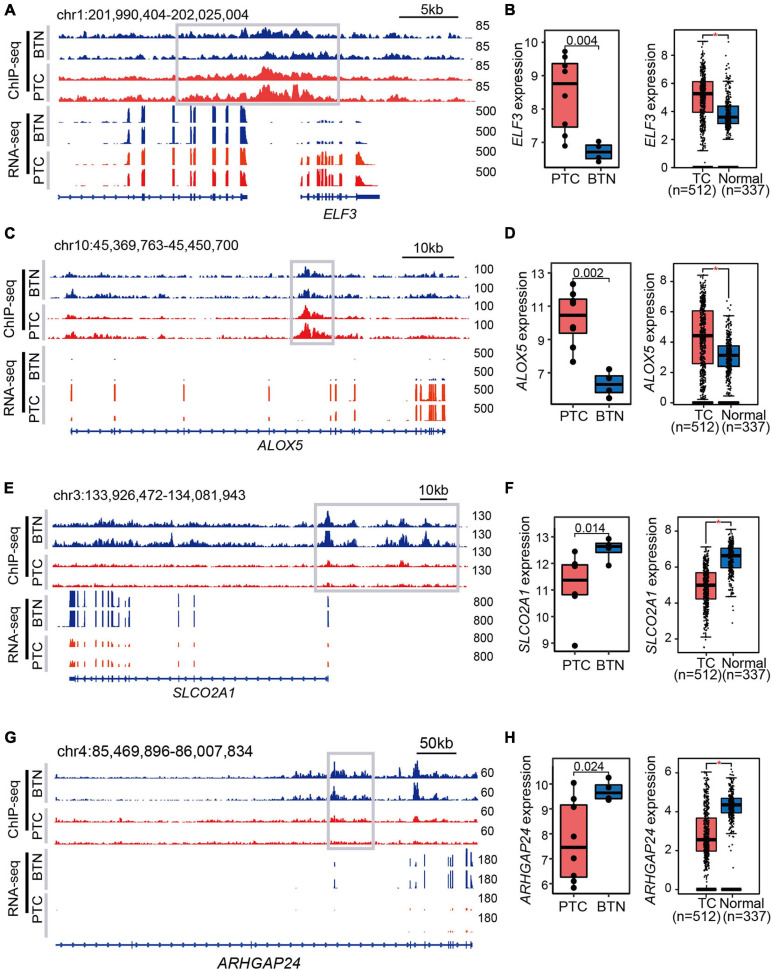
Examples of histotype-specific SE-associated genes. **(A,C)** Gene tracks of PTC-specific SEs. The adjacent genes *ELF3* and *ALOX5* showed strong SE peaks and RNA-seq signals in PTC tissues. **(B,D)** Expression profiles in PTC-specific SEs. Relative expression levels between PTC and BTN are shown in the left panel, while the right box plots indicate expression analysis of TC and normal samples from TCGA and GTEx datasets. **(E,G)** BTN-specific SE tracks and RNA-seq signals. **(F,H)** High expression levels of *SLCO2A1* and *ARHGAP24* in BTN tissues, and similar trends were observed in TCGA and GTEx databases. *p < 0.05. SE, super-enhancer; PTC, papillary thyroid carcinoma; BTN, benign thyroid nodule; TCGA, The Cancer Genome Atlas; GTEx, Genotype-Tissue Expression.

The SEs were assigned to the most adjacent genes, and the expressions of SE-associated genes were higher in the histotype of interest. As exemplified in SE-associated genes in PTC (Figures 4A,C), oncogenes *ALOX5* and *ELF3* were more highly expressed in PTC compared with BTN tissues. Moreover, The Cancer Genome Atlas (TCGA) and Genotype-Tissue Expression (GTEx) data indicated higher levels of *ALOX5* and *ELF3* in TC compared with adjacent and normal thyroid tissues (Figures 4B,D). The tracks of ChIP-seq and RNA-seq signals of *SLCO2A1* and *ARHGAP24* were shown to present wide BTN-specific SEs (Figures 4E,G), coinciding with their higher expressions in BTN and in normal thyroid tissues compared with PTC and TC, respectively (Figures 4F,H). Notably, lower transcription level of *SLCO2A1* was observed in advanced TC stages and associated with poor disease-free survival (Supplementary Figure 1).

### Identification of Genes That Are Sensitive to Epigenetic Drugs and Associate With Prognosis of Papillary Thyroid Carcinoma

**FIGURE 5:**
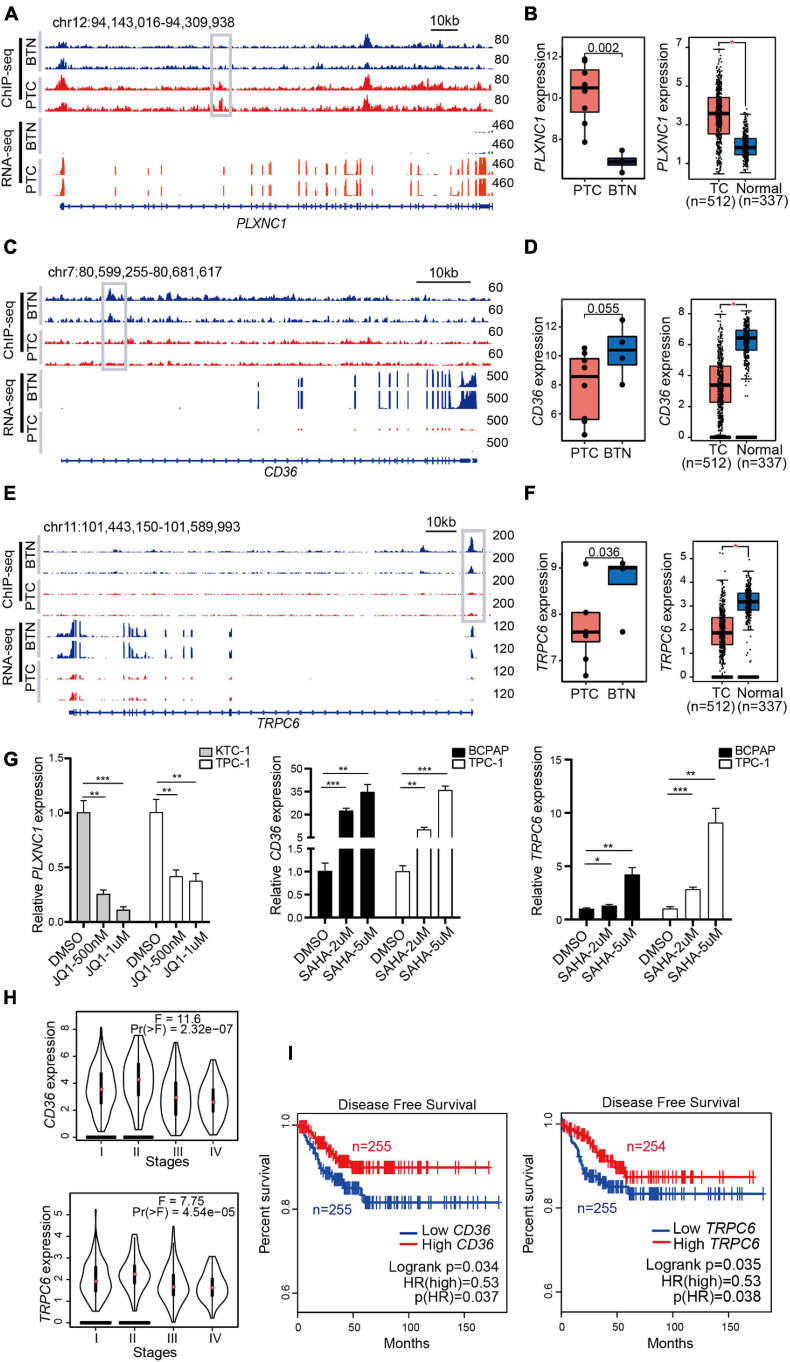
Identification of H3K27ac modification-related genes that are sensitive to epigenetic drugs and associated with the prognosis of PTC. **(A)** H3K27ac ChIP-seq signals and RNA-seq tracks of *PLXNC1*. **(B)** High expressions of *PLXNC1* in PTC (left box plot), and transcriptome data from TCGA and GTEx exhibited consistent difference (right box plot). *p < 0.05. **(C,E)** Strong H3K27ac peaks and RNA-seq signals in *CD36* and *TRPC6* in BTN tissues. **(D,F)** Higher expressions of *CD36* and *TRPC6* in BTN (left box plots) and normal tissues (right box plots). *p < 0.05. **(G)** Treatment with JQ1 or vehicles as control at indicated concentrations for 48 h greatly reduced *PLXNC1* expression levels in KTC-1 and TPC-1 cell lines. SAHA treatment induced a significant higher expression of *CD36* and *TRPC6* in BCPAP and TPC-1 cells. Expression analysis was normalized against GAPDH. *p < 0.05, **p < 0.01, ***p < 0.001. **(H)** Lower levels of *CD36* and *TRPC6* transcription were observed in stage III and IV in TCGA (THCA) datasets. **(I)** Higher levels of *CD36* and *TRPC6* transcription associated with higher disease-free survival rate in patients with thyroid cancers. Analysis was performed with TCGA (THCA) datasets. PTC, papillary thyroid carcinoma; TCGA, The Cancer Genome Atlas; GTEx, Genotype-Tissue Expression.

We integrated H3K27ac ChIP-seq and RNA-seq data to map typical enhancers and promoters to their putative targets. As shown in Figure 5A, PTC samples had stronger H3K27ac signals than BTN samples in the genome location of the *PLXNC1* gene. After a comparison of active chromatin regions of *PLXNC1* with an available database to infer potential enhancers, predicted enhancer–promoter associations were found (Supplementary Figure 2). The RNA-seq revealed that the *PLXNC1* was expressed higher in PTC than BTN samples (Figure 5A). Consistently, a higher expression of *PLXNC1* in PTC than normal controls was found when analyzing data from TCGA and GTEx samples (Figure 5B). In contrast, *CD36* and *TRPC6* showed significantly stronger H3K27ac signals and higher expressions in BTN than PTC (Figures 5C–F). Importantly, treatment of thyroid cancer cells with BRD4 inhibitor JQ1 significantly downregulated the expression of *PLXNC1* and some PTC-specific genes, while the histone deacetylase inhibitors (HDACi) SAHA treatment induced robust increases of *CD36* and *TRPC6* expressions in thyroid cancer cell lines (Figure 5G and Supplementary Figure 3). Furthermore, low expression levels of *CD36* and *TRPC6* were observed in advanced stages of PTC (Figure 5H) and correlated with poor disease-free survival in patients with PTC (Figure 5I), suggesting that these genes are potential predictors for PTC recurrence.

## Discussion

Accumulating evidence suggested that epigenome alterations greatly impact cancer development (Hamdane et al., 2019). In this study, we revealed histone modification features by comparing PTC and BTN, facilitating the knowledge of active regulatory patterns and global expression profiles in thyroid tissues. H3K27ac is an established mark of transcriptional activation. Our data presented differentiated epigenomes between PTC and BTN, with an epigenetic landscape for the PTC progression compared with BTN, and provided references for novel biomarkers for the prognosis of thyroid tumors.

It was recognized that SEs have great impact on tumor pathogenesis and cell identity (Hnisz et al., 2013). The common histone features indicated the common epigenetic modulations between PTC and BTN. Different enhancers might underline specific gene expression patterns in these two groups. In the present study, we found that PTC-specific SE-adjacent genes showed higher expression levels than those in BTN, such as *ALOX5* and *ELF3. ALOX5* had been reported to be highly expressed in PTC compared with normal tissues and was related to PTC tumor invasion (Kummer et al., 2012; Reyes et al., 2019). *ELF3* was reported to be overexpressed with poor prognosis in PTC patients and likely function as an oncogene in TC (Chen et al., 2019). As for BTN-specific SE-adjacent genes identified in this study, *SLCO2A1* was reported to be downregulated in follicular thyroid cancer than benign adenoma (Pfeifer et al., 2013), suggesting its role in thyroid tumor pathogenesis. Several studies reported *ARHGAP24* as a tumor suppressor gene in various malignant tumors, including malignant lymphomas (Nishi et al., 2015), breast cancer (Saito et al., 2012), renal cancer (Wang et al., 2017), lung cancer (Wang et al., 2020), and colorectal cancer (Zhang et al., 2018). However, no study was performed on the functions of *ARHGAP24* in thyroid tumors so far. Though the H3K27ac profile and TCGA database pinpointed that *SLCO2A1* and *ARHGAP24* could be potential predictors for PTC recurrence, their post-transcriptional modification and biological functions require further validation in PTC.

Principal component analysis of PTC and BTN showed two distinct clusters, suggesting etiology-specific epigenetic profiles in these two groups. Interestingly, pathway analysis showed that PTC-specific peaks and genes are enriched in immune-response pathways, indicating that immunoreactions might get involved in thyroid tumor pathogenesis. Whereas the H3K27ac patterns in the two histotypes could not explain all the differentiated gene expressions, one explanation could be that hypomethylation also plays a role in the upregulation of cancer-related genes (Rodríguez-Rodero et al., 2013).

The overexpression of oncogene and silencing of tumor suppressor genes caused by epigenetic modifications were usually reversible with epigenetic enzyme inhibitors. JQ1/SAHA had been assessed in numerous malignancies including TC in clinical trials, and previous studies showed their anti-tumorigenesis functions in TC (Tan et al., 2010). SAHA inhibits deacetylase enzymatic activity. BRD4 inhibitor JQ1 could induce cell growth arrest and apoptosis. The expression levels of certain genes altered following treatment of JQ1 and SAHA, indicating that these drugs might inhibit tumor growth by regulating the expression of some key genes. *PLXNC1* is reported as an oncogene in liver carcinoma (Odabas et al., 2018) and gastric cancer (Chen et al., 2020) and also played a part in TC progression. MiR-4500 repressed PTC development by decreasing *PLXNC1* expression (Li et al., 2019). Besides, it was found that *PLXNC1* was highly expressed in TC (Chen et al., 2013), and the expression level was higher in PTC tissues compared with their paired normal tissues (Qu et al., 2016). Among the genes regulated by SAHA, *CD36* low expression was associated with higher metastasis grade and poor prognosis in colon, breast, and ovarian cancers (Tanase et al., 2020) and pancreatic ductal adenocarcinoma (Jia et al., 2018). *TRPC6* is an important member of the transient receptor potential channels superfamily, participating in the various biological processes (Dietrich and Gudermann, 2014; Jardin et al., 2020). Though it remained unclear if these genes were sufficient to promote or inhibit PTC pathogenesis, their higher transcription levels were related to favorable disease-free rate in TC. Our results suggested the potential roles of these genes in PTC, and their molecular and biological functions warrant further investigation.

While it remained to be elucidated whether epigenetic dysregulation precedes the tumorigenesis, the different histone modification and expression levels in PTC and BTN provided evidence that histone modifications differed in disease states. Though heterogeneity existed in these samples, the results obtained from clustering and statistical algorithm along with supporting evidence from population database and previous biological studies allowed arresting conclusions.

## Conclusion

In summary, our results presented different epigenomic features of PTC and BTN, and dysregulation of enhancers and their targeted genes provide new insights into the pathogenesis and development of PTC. Some enhancer-regulated genes were associated with disease-free survival of PTC, which may hold potential as markers for the prognosis of the disease.

## Data Availability Statement

The original contributions presented in the study are publicly available. This data can be found here: Genome Sequence Archive in National Genomics Data Center, China National Center for Bioinformation/Beijing Institute of Genomics, Chinese Academy of Sciences, under accession number HRA000779.

## Ethics Statement

The studies involving human participants were reviewed and approved by the Institutional Ethics Committee of The First Affiliated Hospital of Sun Yat-sen University. The ethics committee waived the requirement of written informed consent for participation.

## Author Contributions

HX, QZ, RL, and ZT designed the study. WL, JL, YaL, YLi, SH, and SY collected the tissue samples and clinical information. LZ, QL, YLu, XX, and YS performed the experiments. LZ, DX, ZT, YT, XX, RL, and QZ analyzed the data. LZ and DX drafted the manuscript. All authors contributed to the manuscript revision and approved the final version of the manuscript.

## Conflict of Interest

The authors declare that the research was conducted in the absence of any commercial or financial relationships that could be construed as a potential conflict of interest.

## Footnotes

1 http://great.stanford.edu/

2 http://www.webgestalt.org/
