# Supplementary material for: Genome-Wide Histone H3K27 Acetylation Profiling Identified Genes Correlated With Prognosis in Papillary Thyroid Carcinoma
Source: Front Cell Dev Biol. 2021 Jun 11;9:682561. doi: 10.3389/fcell.2021.682561 (PMC8226268; doi:10.3389/fcell.2021.682561)
Supplement: Supplementary file 2 [file Data_Sheet_1.PDF]

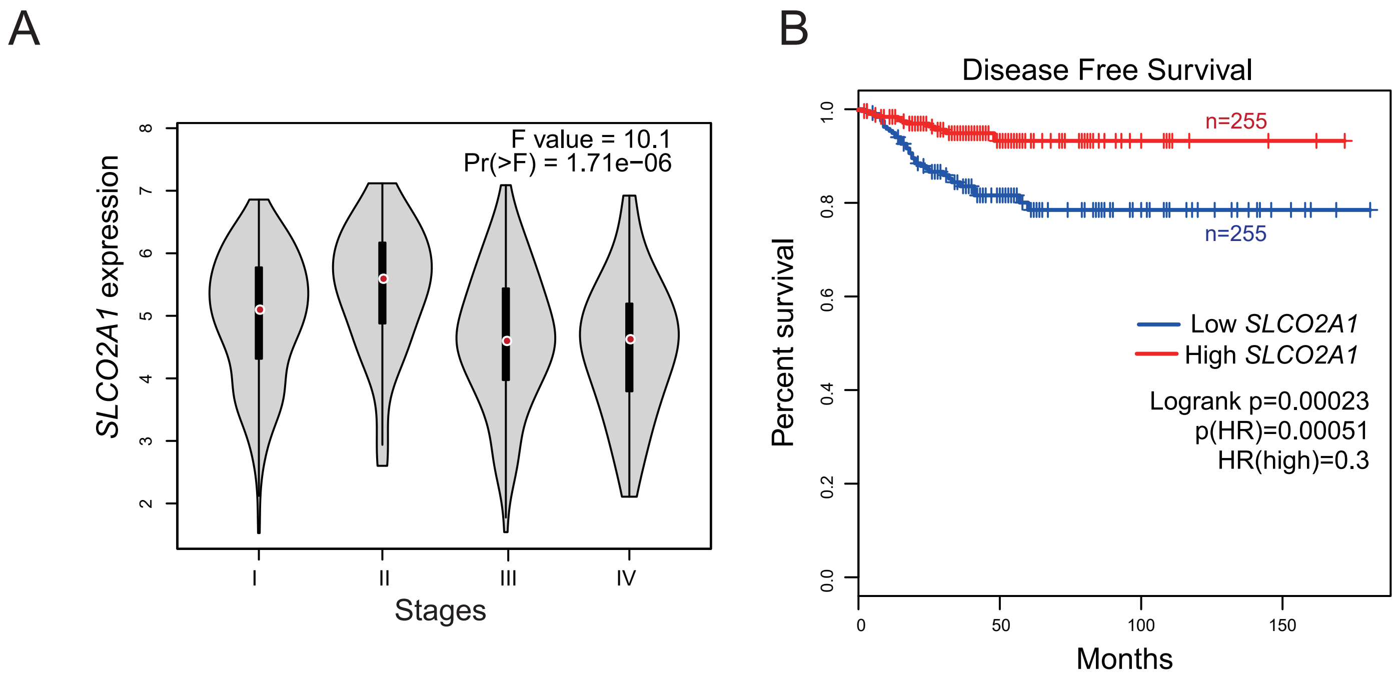

Supplementary Fig.1 The expression and prognostic value of *SLCO2A1* in PTC. (A) Levels of *SLCO2A1* in PTC according to different tumor stages. (B) The association of *SLCO2A1* expression with disease free survival in patients with PTC. Data from the TCGA (THCA) database.

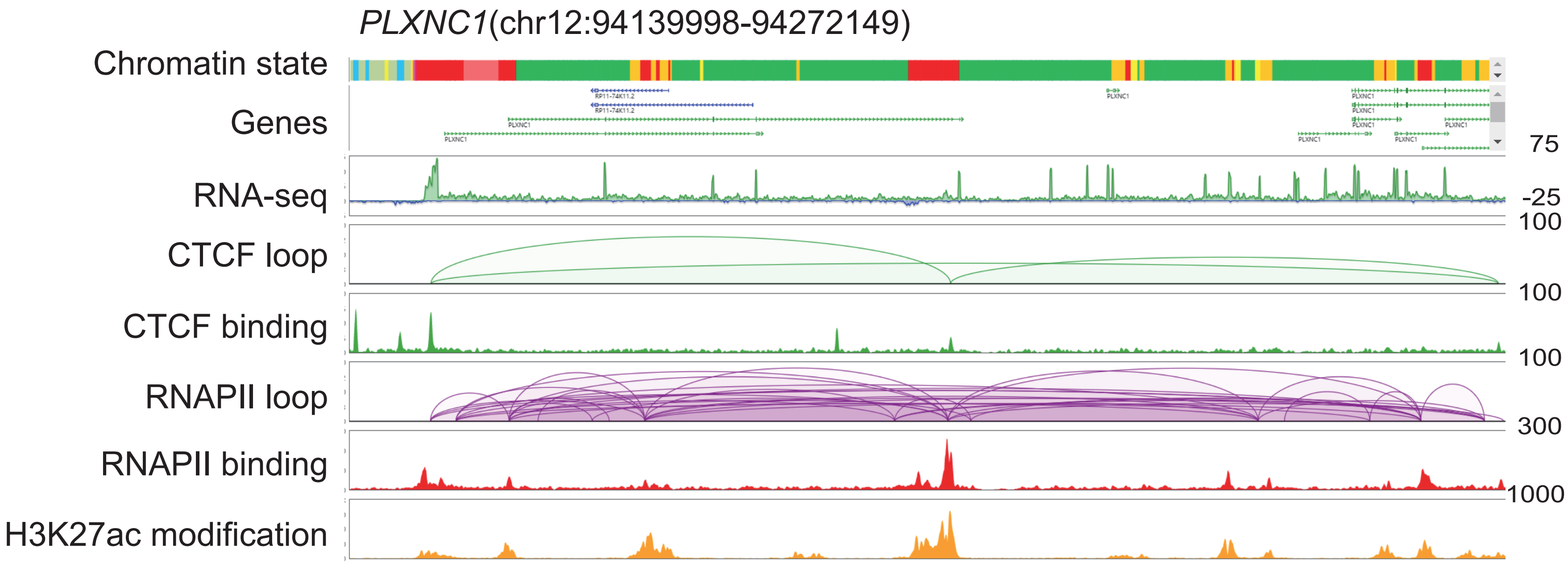

Supplementary Fig. 2 Genomic interactions at *PLXNC1* location in GM12878 cells. Enhancer at the same genomic region with PTC samples was identified in GM12878 cells, looping to *PLXNC1* promoters. Tracks of H3K27ac ChIP-seq, RNA Pol Ⅱ binding and looping, CTCF binding and looping and RNA-seq were shown here.

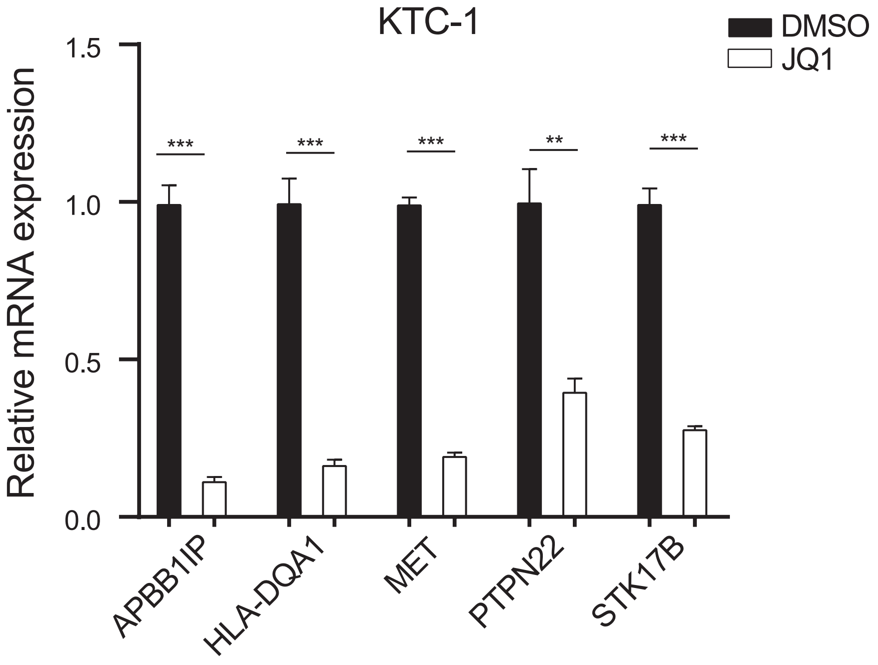

Supplementary Fig.3 Levels of *APBB1IP, HLA-DQA1, MET, PTPN22* and *STK17B* were significantly decreased after treatment with 500nM JQ1 in KTC-1 cells.
